# COVID-19-induced takotsubo cardiomyopathy: Venturing beyond the obvious

**DOI:** 10.1016/j.amsu.2021.102291

**Published:** 2021-04-15

**Authors:** Talal Almas, Tarek Khedro, Abdul Haadi, Reema Ahmed, Lamees Alshaikh, Abdulla Hussain Al-Awaid, Muhammad Siyab Panhwar, Hafeez Ul Hassan Virk

**Affiliations:** a RCSI University of Medicine and Health Sciences, Dublin, Ireland; b Heart and Vascular Institute, Tulane University School of Medicine, New Orleans, LA, USA; c Harrington Heart & Vascular Institute, Case Western Reserve University, University Hospitals Cleveland Medical Center, Cleveland, OH, USA

Takotsubo cardiomyopathy (TCM), colloquially known as “broken heart syndrome,” is a relatively uncommon transient condition that is characterized by left ventricular apical ballooning [1]. It is purported to be triggered by exorbitant physical or emotional stressors. The clinical picture elicited by TCM closely mimics that of acute coronary syndrome (ACS), with most patients presenting with substernal chest pain and ECG changes akin to those observed in a patient with acute myocardial infarction [1]. Interestingly, in cases of TCM, coronary arteries are noted to be unremarkable upon catheterization [1,2]. Due to the remarkably similar clinical semblance elicited by TCM and myocardial infarction, it remains critically imperative to distinguish between the two pathologies. Of note, TCM remains a diagnosis of exclusion, and ischemic cardiomyopathy must be excluded prior to its diagnosis. The diagnosis of TCM is rendered in accordance with the criteria outlined by Mayo Clinic [3]. This is delineated by Fig. 1.

**Fig. 1:**
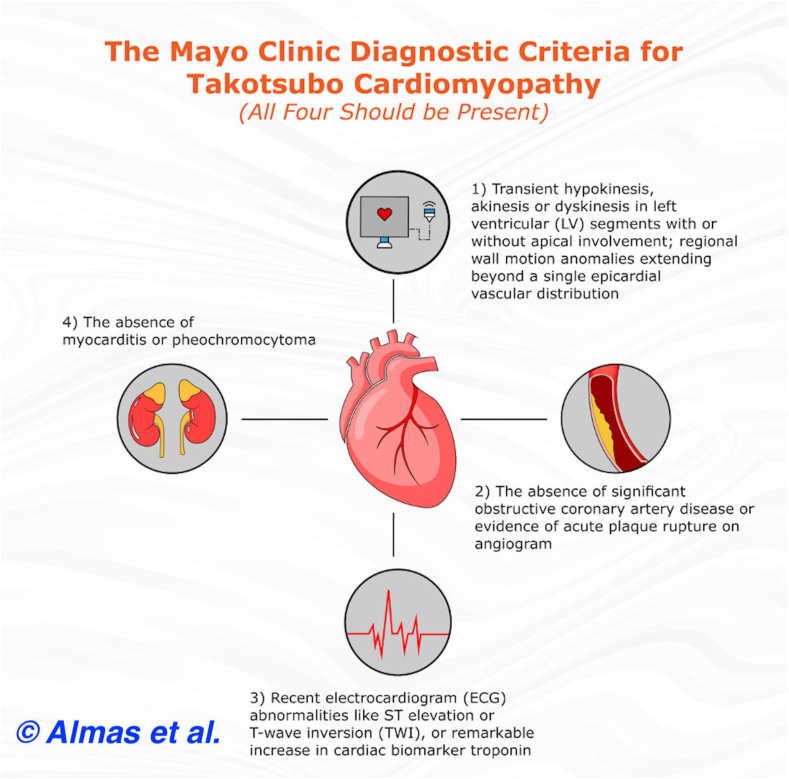
A pictorial depiction of the Mayo Clinic criteria for the diagnosis of takotsubo cardiomyopathy [3].

A systematic review by Gianni et al. divulged that chest pain and dyspnea were the most commonly reported features of TCM [2]. Chest pain was noted to be the chief presenting complaint in 185 of 273 patients (67.8%, 95% CI: 62.0–73.0%; range: 20–94.7%) while dyspnea was noted in merely 40 of 225 patients (17.8%, 95% CI: 13.3–23.3%; range: 4.5–55.5%) who were diagnosed with the ailment. Gianni et al. also established a causal link between TCM and preceding emotional or physical stressors. A prior history of emotional stressors, such as the death of loved ones and insurmountable financial losses, was present in 68 of 254 patients (26.8%, 95% CI: 21.7–32.5%; range: 10–100%) whereas physical stressors including systemic disorders, asthma attacks, and physically-exacting work were found in 96 of 254 patients (37.8%, 95% CI: 32.1–43.9%; range: 14–70%) [2]. Nevertheless, 87 of 212 patients (34.3%, 95% CI: 28.7–40.3%; range: 0–100%) reported an absence of both emotional and physical stressors, indicating that TCM, which is often referred to as “stress-induced cardiomyopathy”, might not always be a sequel of stress [2]. While the medical literature remains largely bereft of studies elucidating a causal relationship between underlying viral infections and TCM, reports surrounding coronavirus disease 2019 (COVID-19) induced TCM have started to surface [[4], [5], [6], [7], [8], [9], [10]]. A brief consideration of the pathophysiology underlying COVID-19 induced takotsubo cardiomyopathy is therefore warranted.

One widely proposed mechanism of COVID-19 induced cardiac injury is directly through the angiotensin converting enzyme 2 (ACE2) receptor, a receptor ubiquitous in the myocardium [4]. The destruction of ACE2 receptors is believed to lead to cardiac injury (1) directly by reducing ACE-2 activity, thereby thwarting the conversion of angiotensin I and II to cardioprotective peptides and (2) indirectly through systemic inflammation and fibrosis [5,6]. Although the underlying pathophysiology of TCM remains at the epicenter of a cardiology conundrum, the transient myocardial changes observed are generally attributed to a surge in catecholamines, such as epinephrine, that is triggered by preceding physical or emotional stress. Furthermore, the observed myocardial changes—the most common of which being apical ballooning of the left ventricle—are transient. Nevertheless, these propositions fail to encompass the mechanisms that underlie COVID-19 induced TCM.

The acute viral illness, severe hypoxia, and the associated acute respiratory distress syndrome (ARDS), as observed in moderate-to-severe COVID-19 infections, can trigger an indirect surge in catecholamines through their sympathetic effects via the adrenergic β1 receptors ubiquitous in the myocardium, culminating in an overactive myocardium [7]. Additionally, modulations in catecholamine sensitivity of the myocardium, mediated by a cytokine storm, are suspected to be critical in the biological and physical manifestations of the disease.

Cytokine storm in the aftermath of a severe SARS-CoV-2 infection has been reported extensively in the literature, with higher levels associated with increased disease severity [4,[6], [7], [8]]. One hypothesis, therefore, would propose two major deleterious effects of the cytokine storm: (1) acting as a physical and chemical stressor in and of themselves as well as (2) increasing catecholamine levels concurrent with an increased hypersensitivity to these catecholamines [11]. Further substantiating this notion is the evidence that elevated levels of catecholamines were discovered to be released locally at the aortic root and the coronary sinus in patients with TCM secondary to COVID-19 [7]. Additionally, patients who have underlying cardiac comorbidities boast higher baseline levels of interleukin-6 (IL-6), tumor necrosis factor-α (TNF-α), and related proinflammatory cytokines, further heralding the onset of TCM [9,11]. Furthermore, COVID-19 can present with a cytokine storm syndrome, which was shown to be associated with a catecholamine surge that may exacerbate pre-existing TCM [9,12,13].

Myocardial stunning due to left ventricular hypokinesis, dyskinesis, or akinesis, which results in a reduced left ventricular ejection fraction, is another proposed mechanism underlying COVID-19 induced TCM [10]. This would support the fact that patients typically present with symptoms akin to those fomented by ACS but without evidence of coronary artery disease or myocyte damage. In one rat model, high dose epinephrine was shown to elicit a cardioprotective response on the myocardium, resulting in a change in the β2 receptor response to that of a G_i_-protein, the consequences of which are negative inotropy [7]. Sufficient alterations to β2 receptors in a segment of the myocardium can lead to akinesis and stunning. Alterations in β1 were also evidenced by biopsy samples performed in vivo: patients with TCM were shown to have higher levels of a G-protein coupled receptor kinase 2 (GRK2) and β-arrestin2 than patients with dilated cardiomyopathy. As GRK2 and β-arrestin2 are known to desensitize the β-adrenergic receptor and β1 and β2 receptors are found together in an apical distribution within the heart, the aforesaid observations could explain the apical ballooning that is seen most frequently in TCM [7].

TCM has also been described in other viral infections. Myocardial involvement has been reported in up to ten percent of influenza cases, with a preponderance of myocarditis cases of varying degrees [14]. One particular case described a 57-year-old woman with an influenza infection and ostensible viral myocarditis [14]. However, given her past history of TCM, the typical TCM ventricular morphology observed through angiogram and echocardiogram, and the restoration of ventricular function, TCM was deemed more plausible. These mechanisms could also portend implications in cases of COVID-19 induced TCM.

In a review of 7 case reports documenting TCM associated with SARS-CoV-2, most patients presented with ECG changes such as ST-segment elevation or T-wave inversion [8,[15], [16], [17]]. Elevated troponin levels were noted in the majority of cases along with reduced left ventricular ejection fraction (LVEF) [4,8,[16], [17], [18], [19]]. Additionally, the echocardiogram divulged underlying apical ballooning, insinuating a diagnosis of TCM. Once identified and managed, most patients showed rapid amelioration in their LVEF, further supporting the diagnosis of SARS-CoV-2-induced TCM. These findings are elucidated by Table 1 below.

**Table 1:** The clinical characteristics and investigative outcomes in patients with COVID-19-induced TCM.

	Taza F^8^	Meyer P^15^	Gomez JMD^4^	Roca E^16^	van Osch D^17^	Sattar Y^18^	Minhas AS^19^
Age	52	83	57	87	72	67	58
Gender	Male	Female	Female	Female	Female	Female	Female
Troponin	normal	Elevated	Elevated	Elevated	Elevated	Elevated	Elevated
ECG	ECG revealed ST segment elevations in the inferior leads	ST-segment elevation + deep T-wave inversions	prolonged QTc interval/sinus tachycardia without ST-wave	negative T waves and repolarization phase alterations + alterations in the left ventricle	negative T-waves and a prolonged QTc interval	new-onset atrial fibrillation with a rapid ventricular response (RVR)	mid-distal left ventricular hypokinesis
	apical ballooning	left ventricular apical ballooning with hyperkinetic basal segments	characteristic echocardiogram findings of apical ballooning	apical ballooning and hypokinesia of the mid-ventricular segments	poor left ventricular systolic function with apical ballooning apical	Diffuse anterior wall and apical akinesia and apical ballooning	Apical ballooning
Estimated left ventricular ejection fraction (LVEF)	45%	Unknown	25%–30%	48%	30%	30%	20%

Lastly, a final proposed stressor that may be overlooked is the emotional and psychological toll caused by the COVID-19 pandemic. In one retrospective study of 1914 patients that presented with acute coronary syndrome during the pandemic, the incidence of stress cardiomyopathy was 7.8% compared to the baseline pre-pandemic incidence of 1.5–1.8%, an incidence ratio of 4.58 (95% CI: 4.11–5.11, p < 0.001) [20]. Notably, all the patients in the study tested negative for SARS-CoV-2 in a reverse transcriptase polymerase chain reaction (RT-PCR) test. As the COVID-19 pandemic continues to wreak havoc, its noxious effects on the emotional and mental health cannot be discounted. While these parameters are more subjective and may not be as easily quantifiable, it is clear that the difficulties inherent in social isolation and “the new normal” are undeniably detrimental to mental health, further increasing the likelihood of developing TCM.

## Ethical approval

NA.

## Funding

NA.

## Author contribution

TA, TK, AH: conceived the idea, designed the study, and drafted the manuscript.

RA, LA, AHA: conducted literature search and created the illustrations.

RA, AHA: revised the manuscript critically and refined the illustrations.

MSP, HUHV: revised the final version of the manuscript critically and gave the final approval.

## Consent

NA.

## Registration of research studies

1. Name of the registry: NA.
2. Unique Identifying number or registration ID: NA.
3. Hyperlink to your specific registration (must be publicly accessible and will be checked): NA.

## Guarantor

Talal Almas

RCSI University of Medicine and Health Sciences

123 St. Stephen's Green

Dublin 2, Ireland

Talalalmas.almas@gmail.com

+353834212442

## Declaration of competing interest

None.
